# Bifid Mandibular Canal: A Proportional Meta-Analysis of Computed Tomography Studies

**DOI:** 10.1155/2023/9939076

**Published:** 2023-03-06

**Authors:** Nyan Min Aung, Kyaw Kyaw Myint

**Affiliations:** Department of Oral Biological Science, University of Dental Medicine, Mandalay, Mandalay Division, Myanmar

## Abstract

**Introduction:**

Growing body of evidences showed different grades in prevalence of bifid mandibular canals. Because the previous reviews focused solely on patient-level occurrence, hemi-mandible-level prevalence, bilateral symmetry, length, and diameter of bifid mandibular canals were required to be estimated collectively. The research question of this meta-analysis was “What is the prevalence of bifid mandibular canal among patients seeking computed tomography examinations”?

**Materials and Methods:**

In vivo, computed tomography, and cross-sectional studies were eligible. Studies, with less than 100 subjects or anatomic site restriction or controlled class of bifid mandibular canal, were excluded. Joanna Briggs Institute (JBI) critical appraisal tool for prevalence studies was used to assess methodological quality of all included studies. Random effect meta-analyses for proportion of bifid mandibular canal were done.

**Results:**

40 studies met the inclusion criteria. All studies were selected for both systematic review and meta-analyses. Totally, 17714 patients and 31973 hemi-mandibles were included. All eligible studies showed moderate risk of bias on average. Resulting from the random effect model, more than 20% of patients seeking computed tomographic examinations had bifid mandibular canals (BMCs) which penetrated into slightly more than 14% of hemi-mandibles. Of the patients having bifid mandibular canals (BMCs), nearly 23% exhibited such anatomy on both sides of their mandibles. Estimated mean length and diameter of the accessory canals of bifid mandibular canals were 12.17 mm and 1.54 mm, respectively.

**Conclusion:**

The geographical locations, classifications, reliability test, and voxel size of computed tomography were all implicated in the prevalence of bifid mandibular canals along with gender and laterality, although considerable heterogeneity and bias were detected.

## 1. Introduction

**Figure 1:**
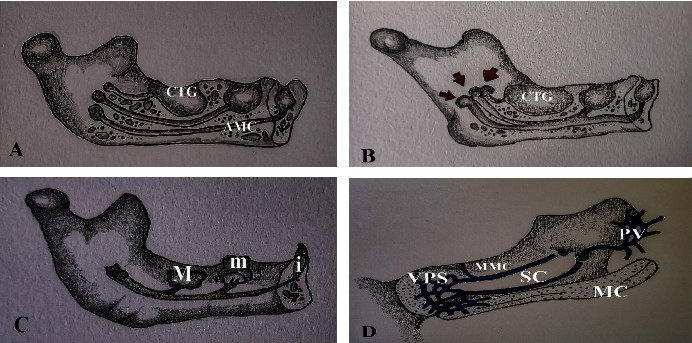
Developmental theories of mandibular canal. Hypothesis of Chaves Lomeli: (a) three separate accessory mandibular canals (AMC) draining into corresponding bony crypt of tooth germ (CTG); (b) red arrows indicate fusion of three separate accessory mandibular canals; (c) after fusion, formation of a single mandibular canal draining deciduous mandibular incisor (i), deciduous second mandibular molar (m), and permanent mandibular first molar (M). Investigation of Serres: (d) vein in Serres canal (SC) draining into pterygoid venous plexus (PV) and venous plexus (VPS) at symphysis cartilage along with Meckel's cartilage (MC), paralleling to main mandibular canal (MMC) with vein.

The formation of bifid mandibular canal (BMC) originates from two fundamental theories. More recent hypothesis was inspired from the investigation of Chaves Lomeli and colleagues [1]. They speculated that bifurcation of mandibular canal may be molded by partial fusion of three accessory mandibular canals (Figures 1(a)–1(c)). They confirmed that these three canals were supplying tooth germs of mandibular deciduous incisors, deciduous ,molars and permanent first molars in the mandible of human fetus. However, they did not mention how these canals fused with each other.

Another one, a historical finding, was explored by observation of Serres. This French embryologist examined the vein, in one additional mandibular canal, draining below main canal [2]. This canal was confirmed by one recent cadaver report [3], but the authors did not report it as Serres's canal. Also, one human embryonic study [4] corroborated the existence of Serres's vein draining both pterygoid venous plexus posteriorly and venous plexus at mandibular symphysis anteriorly along with Meckel's cartilage (Figure 1(d)) of human embryo. Unfortunately, all these studies never stated where the mechanism of forming such anomaly came from.

Prior to forming BMCs, there are numerous amounts of anatomical variations along the extra-osseous course of inferior alveolar nerve (IAN) branching pattern. Proximally, IAN was found entrapped in the muscle fibers of inferior head of lateral pterygoid muscle [5]. Occasionally, it attained neural anastomosis with lingual nerve, long buccal nerve, auriculotemporal nerve, retromolar nerve, and mylohyoid nerve [6] before entering into the mandibular foramen. Also, at this entrance, it may seldom be penetrated by the maxillary artery [7]. In addition, progressive bone resorption in the edentulous mandible may expose main mandibular canal [8] and accessory lingual canal in the anterior mandible [9].

Subsequently, the mental nerve, an intra-osseous branch of IAN, could encroach anteriorly from its branching point. Thereby, it turns backwards and exits through the mental foramen, forming a loop called anterior loop [10]. In coronal section of CBCT, it can be seen as a numerical “8” character. Around a mental foramen, the existing mental nerves may be accustomed to multiple openings called accessory mental foramina [11]. The most common position of these foramina was documented at the location posterior and inferior to the main mental foramen [11]. So, along the route of IAN, several anatomical variations can take part as a series of events. These may become associated with unavoidable clinical complications during oral surgical procedure.

Inadequate local anesthesia of IAN can be associated with presence of BMC [12]. This aberrant anatomy was also found in fifteen percent of the patients with postoperative neurological disturbance after mandibular third molar extraction [13]. Interestingly, one systematic review figured out that BMC was seldom entrapped between the roots of mandibular third molar [14]. In this case, a more complex treatment option was needed to be planned to undergo third molar extraction. Surprisingly, inferior alveolar nerve was thought to have neurological anastomosis with long buccal nerve through retromolar canal [6]. So, infrequently, aberrant long buccal nerve was investigated during last molar surgery [15]. Hypoesthesia, partial or total loss of sensation, of buccal gingiva was found to be an unpleasant consequence of such procedure. Presence of neurovascular bundles in retromolar type of BMC was confirmed by one cadaver study [16]. Occasionally, inferior alveolar neurovascular bundle was recorded to become injured by endodontic procedure, implant installation, and osteotomy procedure [12]. As a result, we should keep in mind that anatomical variations of inferior alveolar nerve should be identified preoperatively with a proper investigation method.

In comparison with and stating cone beam computed tomography (CBCT) as a reference standard, sensitivity of orthopantomogram (OPG), in detecting BMCs, ranged from 11% to 76% [13, 17, 18]. By defining magnetic resonance imaging (MRI) as reference gold standard, only 16.67% of BMCs were found in CBCT [19]. Sometimes, bone marrow [20], instead of vein, artery, and nerve (VAN) assembly, was also observed in the accessory canal of BMC in histological section.

Several numbers of systematic reviews and meta-analysis showed different grading in prevalence of BMCs. At patient-level prevalence of BMCs, Shan and coworkers recently investigated 38% [21]. Valenzuela and associates analyzed 57% of this anomaly at individual level [22]. Also, Hass and colleagues identified 16.25% of this aberrant anatomy at patient level [23]. One literature review stated that BMC ranged between 0.05% and 69% of the population [12]. In the review of Shah and Mehta [24], retromolar canal, one type of BMCs, revealed 3.2% to 93.5% of dry mandibles. Castro and fellows [25] contributed classifications of BMCs. They focused mainly on the radiographic methods of current classifications. However, units of analysis, such as patient-level or hemi-mandible-level prevalence of BMCs, and similarity of different classifications were not considered in such review. Nearly all the reviews included had a wide variety of research methodology, not objectively on computed tomographic examination.

Mostly, these reviews comprised especially patient-level incidence of BMCs. Hemi-mandible-level occurrence, bilateral symmetry, length, and diameter of this variation were required to be noted and pooled proportionally. Also, laterality, sexual dimorphism, prevalence across different continents, and different classifications of such variation were still questioned to be found as combined effect size (pooled proportion). Additionally, we also needed to know how reliability test before computed tomography examination and voxel size of CBCT were influencing the prevalence.

Finally, the question of this meta-analysis was “What is the prevalence of bifid mandibular canal (BMC) among patients seeking computed tomography examinations”?

The objectives were

- To observe patient-level prevalence of BMCs
- To find hemi-mandible-level prevalence of BMCs
- To identify bilateral symmetrical distribution of BMCs
- To estimate mean length and diameter of BMCs

## 2. Materials and Methods

### 2.1. Selection Criteria

#### 2.1.1. Types of Primary Studies

The eligible primary studies were as follows:

1. Studies conducted on living humans (in vivo)
2. The images were obtained from cone beam computed tomography (CBCT) or computed tomography (CT) or multi-slice computed tomography (MSCT) or spiral computed tomography (SCT) or multi-detector computed tomography (MDCT) or the combination of these methods
3. Cross-sectional study design
4. Descriptive or analytical study design in comparison with orthopantomograph (OPG) or other research methods
5. Prospective or retrospective

#### 2.1.2. Types of Excluded Studies

The studies which were eligible for exclusion:

1. BMC restricted to the specific anatomic location, for example, solely focusing on mandibular ramus or body
2. Only the controlled category of BMC, for example, strictly to record retromolar canal or coronoid canal regardless of other types of BMCs
3. The correlation studies between the presence of BMC and a confounding factor such as inflammation
4. Living human sample <100 due to lower generalizability
5. Studies which used unclear working definition of BMC
6. Studies used other research methods, for example, in vitro or OPG
7. Studies which discovered dimension, location, position, and course of main mandibular canal
8. Studies which investigated only the regions of mandibular third molars and implant sites
9. Case reports, case series, literature reviews, conference paper, systematic reviews and meta-analyses, book chapters, letters to editors, opinion, commentary, secondary data analysis, and comparative dental anatomy

#### 2.1.3. Outcomes of Interest

Because of being categorical variable, numerators (outcomes of interest) were defined as

1. Number of patients with BMCs (objective Ι)
2. Number of hemi-mandibles with BMCs (objective ΙΙ)
3. Number of patients with bilateral symmetrical distribution of BMCs (objective ΙΙΙ)

Length and diameter of BMC in millimeters (objective ΙV) were stated as continuous variables.

#### 2.1.4. Population

Patients with no age limitation, no history of trauma and pathologies at the mandible, and no record of orthognathic surgery and bone graft were included.

Denominators were stated as

1. Total number of patients (objective Ι)
2. Total number of hemi-mandibles (objective ΙΙ)
3. Total number of patients with BMCs (objective ΙΙΙ)

### 2.2. Literature Search

The search was focused mainly on various terms of conditions and context. The search terms were as follows.

*Conditions*. Bifid Mandibular Canal, BMC, Bifid canal, Bifurcated Mandibular Canal, Mandibular canal, MC, Inferior Alveolar Canal, IAN, Inferior Dental Canal, Mandibular Canal Bifurcation, Variations of Mandibular Canal, Double Mandibular Canal, Accessory Mandibular Canal, Mandibular Canal Branch, Branching of Mandibular Canal, Branched Mandibular Canal, and Accessory Mandibular Canal.

*Contexts*. CBCT, Cone Beam Computed Tomography, Cone Beam CT, Computed Tomography, Multi-detector Computed Tomography, MDCT, Multi-slide Computed Tomography, MSCT, Spiral Computed Tomography, and SCT.

The search was accommodated in the frame of “1 AND 2”. PubMed, Google Scholar, ResearchGate, ProQuest, Scopus, and LILACS were all explored. There was no language and time restriction. Search procedures were carried out from inception to April 2022. Back searching was done through the citation lists of the articles. Authors of eligible studies were contacted via ResearchGate. We created Gmail alert for similar articles in Google Scholar during the period of literature search.

### 2.3. Data Collection

We approached the data from each individual study: total number of dental patients, hemi-mandibles, patients with BMCs, and hemi-mandibles with BMCs, bilateral symmetrical distribution of BMCs, mean lengths and diameters of BMCs along with their standard deviations (SDs), gender, geographical locations, country, sampling frame, sample size calculation, randomization or consecutive series or convenience sampling of patients, reliability test, population coverage, adequacy of outcome reporting, types of study design, conditions (outcome of interest) defined in primary studies, types of computed tomography, its voxel size, field of view (FOV), mA (milliampere), and kVp (kilo voltage).

### 2.4. Assessment of Methodological Quality

We investigated the research methods of the included studies using the Joanna Briggs Institute (JBI) critical appraisal tool for systematic reviews of prevalence studies.

9 questions were included in the appraisal. 9^th^ question of these was excluded. As a consequence, eight questions were retained.

Then, we categorized the identified articles into three subgroups: JBI score (8, 7, and 6), (5 and 4), and (≤3). JBI 8, 7, and 6 were consistent with low risk of bias, 5 and 6 were consistent with moderate risk of bias, and less than or equal to 3 was consistent with high risk of bias [26]. The percentage of JBI score gained by each category was calculated by the following formula: (the summation of JBI scores obtained from each study/total JBI scores) × 100. Finally, average JBI score of all included studies was estimated.

Subsequently, the research methodology of all included studies contributed to chart about the domains of frame of sampling, calculation of sample size, methods of sampling (convenience, randomization, and consecutive sampling methods), reporting of setting detail, and reliability measure before computed tomography examination, validity of measurement instrument, coverage of sample, and completeness of outcome reporting.

### 2.5. Statistical Analysis

Three formulas [26] for the corresponding objectives (Ι, ΙΙ, and ΙΙΙ) were (number of patients with BMCs/total number of patients) × 100 for objective Ι, (number of hemi − mandibles with BMCs)/(total number of hemi − mandibles) × 100 for objective ΙΙ, and (number of patients with bilateral presence of BMCs/total number of patients with BMCs) × 100 for objective ΙΙΙ.

The abovementioned numerator and denominator variables were put into Excel spreadsheet of MetaXL software to undergo meta-analysis by the random effect model.

Standard errors (SEs) for mean lengths and diameters (objective ΙV) of BMCs were calculated by the following formula [27]: $SE=\left(SD/\sqrt{n}\right)$, where SD = standard deviation and *n* = sample size. Then, mean lengths and diameters together with their corresponding SEs and number of observations were all put into an Excel spreadsheet of Meta-Essentials software to meta-analyze. The generic inverse variance method and random effect model were used for such analysis [27].

To explore heterogeneity, subgroup analyses by the random effect model were conducted through male versus female, right versus left, patient-level prevalence of BMCs across different continents, hemi-mandible-level prevalence of BMCs across different continents, patient-level and hemi-mandible-level prevalence of BMCs among the different classifications, Naitoh's classification, Norje's Classification, and Langlais's classification, and BMC with two mandibular foramina (Langlais ΙV or Norje ΙΙΙ). Heterogeneity was measured with *I*^2^ statistic for proportion of BMC [27].

Sensitivity analysis by random effect model was done by excluding the studies which did not undertake reliability test before CBCT examinations. The rest of the studies with calibration test were subjected to meta-analysis again.

Moderator analysis was carried out by correlating the voxel of CBCT in millimeters and prevalence of BMCs. For this analysis, standard errors (SEs) for proportions of BMCs were calculated by the following equation [27]: $SE=\sqrt{\left(P\left(1-P\right)/n\right)}$, where *p* = proportion of BMCs and *n* = sample size. Then, proportions of BMC prevalence, their corresponding SEs, number of observations, and values of voxel size (moderator) were put together into Excel spreadsheet of Meta-Essentials software to be meta-analyzed. Regression lines were drawn, and the random effect model was used for this analysis.

### 2.6. Publication Bias Test

Publication bias tests were performed through objectives Ι, ΙΙ, ΙΙΙ, and ΙV by inspecting funnel plots. *X*-axis of the plot was arcsine prevalence and *Y*-axis was standard error for objectives Ι, ΙΙ, and ΙΙΙ. For objective ΙV, *X*- and *Y*-axes were effect sizes (mean length or diameter) and standard error.

For objectives Ι, ΙΙ, and ΙΙΙ, visual inspection of funnel plot asymmetry was justified by Doi plot and LFK index [28]. Doi illustrates “no asymmetry” concerning with lack of publication bias, “minor asymmetry” indicating small amount of bias, and “major asymmetry” confirming presence of publication bias. ±1 LFK index reveals the certainty of publication bias [28].

For objective ΙV, funnel plot was intended to be repaired by the trim-and-fill method [29]. This reveals how many studies are needed to be filled to neutralize pooled effect size when asymmetry (publication bias) is present.

All the analyses were accomplished in MetaXL and Meta-Essentials software.

### 2.7. Commonality among Classifications and Working Definitions of BMC

Some differences can be seen among Naitoh's, Norje's, and Langlais's classifications [30−32]. Fortunately, commonality among these classifications was graphed in Table 1.

#### 2.7.1. Working Definitions of BMCs

  Bifid mandibular canal is defined as the mandibular canal with a branch originating from its trunk in either sagittal or coronal sections of three-dimensional radiographs.
  Forward canal: two canals, from one mandibular foramen, branching front with the absence of joining.
  Dental canal: an accessory canal, branching from main mandibular canal, supplying permanent mandibular first, second, or third molars.
  Buccolingual canal: from mandibular canal, a branch orienting in buccal or lingual direction, only explained in coronal section of CBCT image.
  Retromolar canal: one accessory canal, from the main inferior alveolar canal, distributes at or around retromolar region.
  Forward confluent canal: forward accessory canal rejoining into its main mandibular canal.
  Two mandibular foramina: two mandibular canals, originating from separate mandibular foramina, merging in the body of the mandible.

The extensions of Naitoh's classification, which were not included in Table 1, were as follows. Inferior bifid canal (bicanal): an accessory canal branching inferior from main mandibular canal and then running forward.

TMC (trifid mandibular canal) was not counted for the meta-analysis when primary studies had reported both BMC and TMC.

## 3. Results

**Figure 2:**
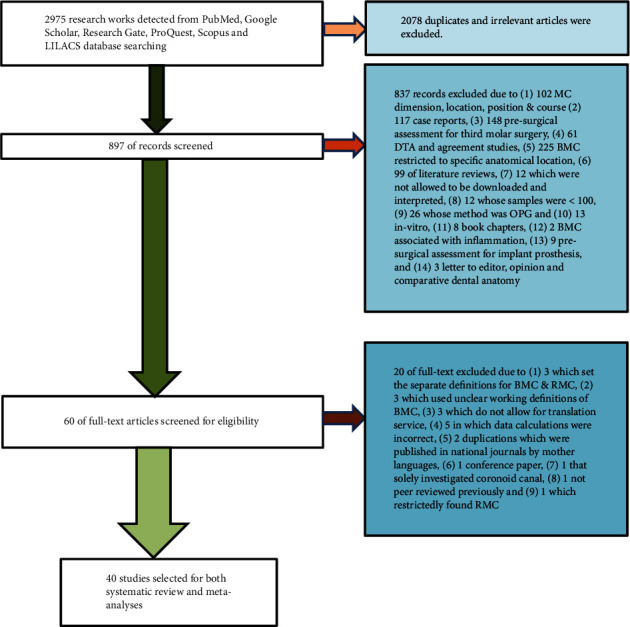
Flow diagram of screening and processing the studies.

All stages of identifying and selecting the records were illustrated in the flow diagram (Figure 2).

Forty studies [13, 18, 33–70], which met the eligibility criteria, were chosen for both methodological quality assessment and quantitative meta-analyses.

One study displayed JBI score of 8/8 [36], 4 studies had a JBI score of 7/8 [35, 37, 50, 58], 3 studies displayed JBI score of 6/8 [42, 44, 52], 7 studies had a JBI score of 5/8 [34, 40, 41, 49, 56, 64, 70], 13 studies had a JBI score of 4/8 [33, 39, 43, 46, 47, 53–55, 59, 60, 63, 65, 67], 10 studies had a JBI score of 3/8 [13, 18, 38, 48, 57, 61, 62, 66, 68, 69], and 2 studies had a JBI score of 2/8 [45, 51]. As a result, 8 studies had a mean JBI score of 84.37%, having low risk of bias. 20 studies had the average score of 54.38%, comprising moderate risk of bias. The remaining 12 had an average JBI of 34.37%, meaning high risk of bias. Overall mean JBI score of all eligible studies was 54.69% demonstrating moderate risk of bias.

**Figure 3:**
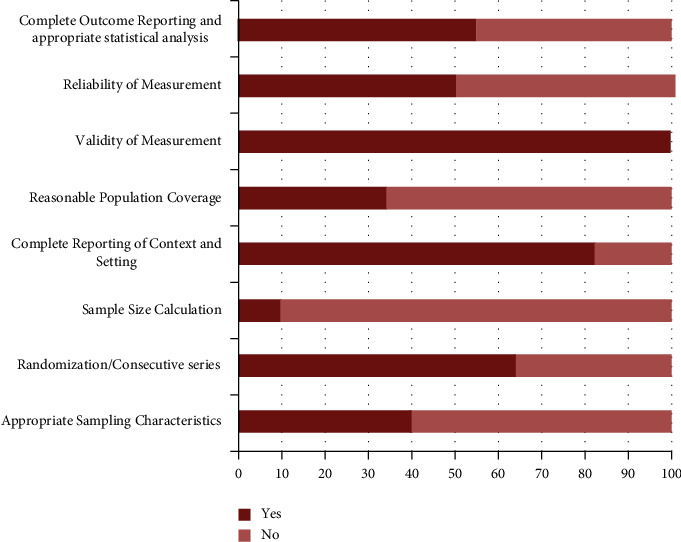
Reported methodology of the included studies.

The detected research methodology of all included studies is summarized in Figure 3.

Population, country, number of patients with genders, number of hemi-mandibles, age of the patients, geographic location, various definitions of BMC, settings, and study design of the eligible studies are described in Table 2.

Totally, 17714 patients were identified from the included studies of the review. 6475 males and 7947 females were reported. 31973 hemi-mandibles were found for this review. Age of the patients ranged from 6 to 103 years.

### 3.1. Patient-Level Prevalence of BMCs

Thirty six studies [33–62, 64–68, 70] revealed patient-level prevalence of BMC. The total number of dental patients in the meta-analysis was 17239 of which 2985 had BMCs.

**Figure 4:**
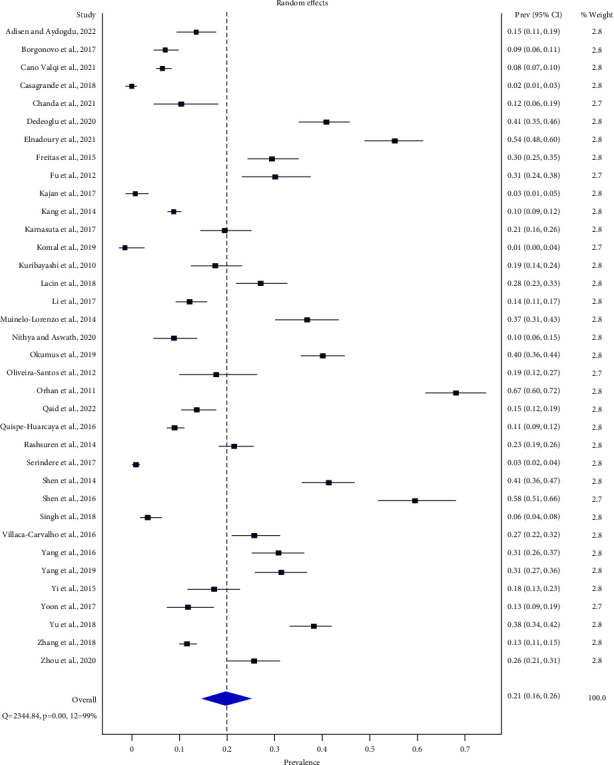
Forest plot of patient-level prevalence of BMC.

The pooled patient-level prevalence of BMC was 20.7% (95% CI: 15.9%–26%) (range: 1%–67%) (*Q* = 2344.84, *p* < 0.05, *I*^2^ = 99%) by the random effect model (Figure 4).

### 3.2. Hemi-Mandible-Level Prevalence of BMCs

Thirty eight studies [13, 18, 33–45, 47–67, 69, 70] demonstrated hemi-mandible-level prevalence of BMCs. The total number of hemi-mandible in this meta-analysis was 31603. BMC was found in 3846 of these hemi-mandibles.

**Figure 5:**
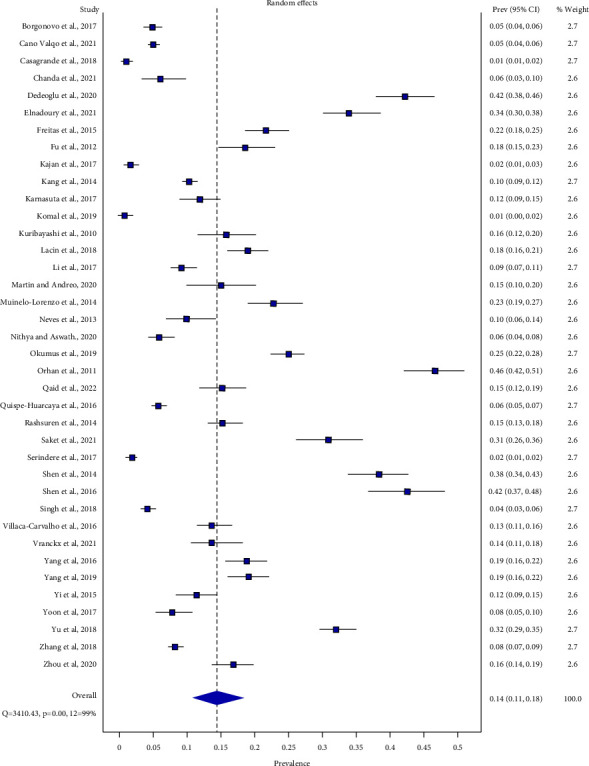
Forest plot of hemi-mandible-level prevalence of BMC.

The summarized hemi-mandible-level prevalence of BMC was 14.3% (95% CI: 10.7%–18.3%) (range: 1%–46%) (*Q* = 3410.43, *p* < 0.05, *I*^2^ = 99%) by the random effect model (Figure 5).

### 3.3. Bilateral Symmetrical Distribution of BMCs

Twenty nine studies [35–39, 42–46, 48–52, 54–62, 64–67, 70] displayed bilateral symmetrical distribution of BMC. The total number of dental patients in this analysis was 2416. 697 of these patients had BMCs on both sides of their mandibles.

**Figure 6:**
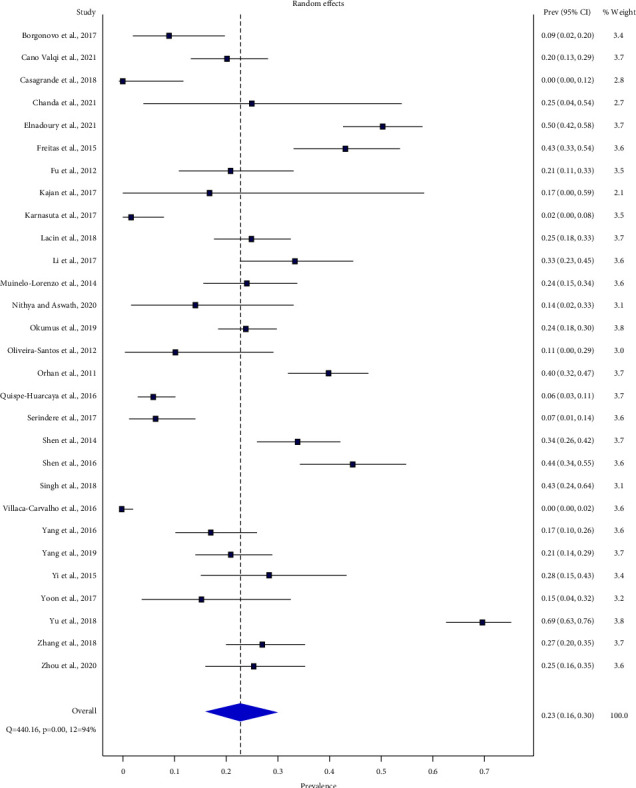
Forest plot of bilateral symmetrical distribution of BMC.

The pooled bilateral symmetrical distribution of BMC was 22.8% (95% CI: 16.3%–30%) (range: 0%–69%) (*Q* = 440.16, *p* < 0.05, *I*^2^ = 94%) in the random effect model (Figure 6).

### 3.4. Mean Length and Diameter of BMCs

Ten studies [33, 37, 40, 41, 43, 50−52, 63, 70] reported the mean lengths of accessory canal of BMCs. The total number of accessory canals of BMCs in the meta-analysis was 1091. The estimated mean length of the accessory canal was 12.14 mm (95% CI: 10.08 mm–14.21 mm) (SE (standard error): 0.91, 95% PI (prediction interval): 6.65 mm–17.64 mm) (range of mean lengths: 7.1 mm–16.9 mm).

Thirteen studies [33, 36, 37, 40, 41, 43, 46, 47, 50–52, 67, 70] displayed the mean diameters of accessory canal of BMCs. The total number of accessory canals of BMCs in this analysis was 1278. The estimated mean diameter of this accessory canal was 1.54 mm (95% CI: 1.27 mm–1.82 mm) (SE: 0.12, 95% PI: 0.64 mm–2.45 mm) (range of mean diameters: 0.9 mm–2.28 mm).

### 3.5. Subgroup Analysis

#### 3.5.1. Male versus Female

Twenty five studies [33, 35, 37–45, 47, 49–52, 54, 56, 57, 60–61, 64, 66, 68, 70] figured out patient-level prevalence of BMC according to gender. Total male patients in the analysis were 4933 of which 1001 had BMCs. Total female patients were 6397 of which 1074 had BMCs.

The pooled patient-level prevalence of BMC, in both male and female, was 22.6% (95% CI: 17.5%–28.1%) (range: 3%–67%) (*Q* = 466.67, *p* < 0.05, *I*^2^ = 95%) and 18.9% (95% CI:14%–24.2%) (range: 2%–66%) (*Q* = 634.72, *p* < 0.05, *I*^2^ = 96%) by the random effect model.

As a result, BMC was significantly found in male patients than females (chi-square statistic: 15.7143, *p* value = 0.000074).

#### 3.5.2. Right versus Left

Twenty three studies [36, 37, 39, 42–45, 49–52, 55–58, 60–61, 64–67, 69, 70] identified hemi-mandible-level prevalence of BMCs according to sides of mandible. The total number of right hemi-mandibles in the analysis was 11417 of which 1228 had BMCs. The total number of left was 11411 of which 1112 had BMCs.

The estimated right and left hemi-mandible-level prevalence of BMC was 14% (95% CI: 9.3%–19.4%) (range: 1%–50%) (*Q* = 1297.027, *p* < 0.05, *I*^2^ = 98%) and 12.6% (95% CI: 8.6%–17.2%) (range: 1%–43%) (*Q* = 1011.544, *p* < 0.05, *I*^2^ = 98%) by the random effect model.

As a result, BMC was more investigated on the right side of mandible than in the left predominantly (chi-square statistic: 5.1607, *p* value = 0.023103).

#### 3.5.3. Patient-Level Prevalence of BMCs across Different Continents

In terms of the patient-level prevalence of BMC, from highest to lowest, European population demonstrated 26.5% (95% CI: 10.6%–46.1%) (range: 3%–67%) (*Q* = 1083.72, *p*=0.0001, *I*^2^ = 99%), Asian population demonstrated 18.8% (95% CI: 14.1%–24%) (range: 1%–58%) (*Q* = 742.96, *p*=0.0001, *I*^2^ = 97%), and American population demonstrated 13.9% (95% CI: 7.5%–21.7%) (range: 8%–30%) (*Q* = 241.62, *p*=0.0001, *I*^2^ = 98%), respectively.

There were no data to pool the estimates for both African and Australian populations.

#### 3.5.4. Hemi-Mandible-Level Prevalence of BMCs across Different Continents

In terms of the hemi-mandible-level prevalence of BMC, African population displayed 32.8% (95% CI: 29.6%–36.1%) (range: 31%–34%) (*Q* = 1.087, *p*=0.297, *I*^2^ = 8%), European population displayed 17.2% (95% CI: 7.2%–30.1%) (range: 2%–46%) (*Q* = 1554.73, *p*=0.0001, *I*^2^ = 99%), Asian population displayed 13.3% (95% CI: 9.4%–17.7%) (range: 1%–42%) (*Q* = 940.68, *p*=0.0001, *I*^2^ = 98%), and American population displayed 7.8% (95% CI: 4%–12.7%) (range: 1%–21%) (*Q* = 302.52, *p*=0.0001, *I*^2^ = 98%) in descending order.

There were not enough data to summarize the values for Australian population.

#### 3.5.5. Patient-Level and Hemi-Mandible-Level Prevalence of BMCs among the Different Classifications (Naitoh's, Norje's, and Langlais's Classifications)

Patient-level prevalence of BMCs was 23.9% (95% CI: 18.1%–30.3%) (range: 3%–67%) (*Q* = 1890.696, *p*=0.0001, *I*^2^ = 97%) in Naitoh's classification, 17.7% (95% CI: 13.7%–21.9%) (range: 13%–21%) (*Q* = 4.103, *p*=0.129, *I*^2^ = 51%) in Norje's classification, and 2.9% (95% CI: 1.3%–5%) (range: 1%–6%) (*Q* = 11.192, *p*=0.011, *I*^2^ = 73%) in Langlais's classification, respectively. The prevalence was significantly more common in Naitoh's classification than Norje's and Langlais's classifications (chi-squared statistic: 153.0513, *p*value <0.00001).

Hemi-mandible-level prevalence of BMCs was 16.9% (95% CI: 12.1%–22.2%) (range: 2%–46%) (*Q* = 2813.873, *p* < 0.05, *I*^2^ = 99%) in Naitoh's classification, 11.3% (95% CI: 7.2%–16.1%) (range: 8%–16%) (*Q* = 11.495, *p*=0.003, *I*^2^ = 83%) in Norje's classification, and 1.6% (95% CI: 0.4%–3.4%) (range: 1%–4%) (*Q* = 23.659, *p*=0.0001, *I*^2^ = 87%) in Langlais's classification, respectively. The prevalence was enormously more investigated in Naitoh's classification than Norje's and Langlais's classifications (chi-squared statistic: 251.8578, *p* value = 0.00001).

#### 3.5.6. Naitoh's Classification (Hemi-Mandible-Level Prevalence)

**Figure 7:**
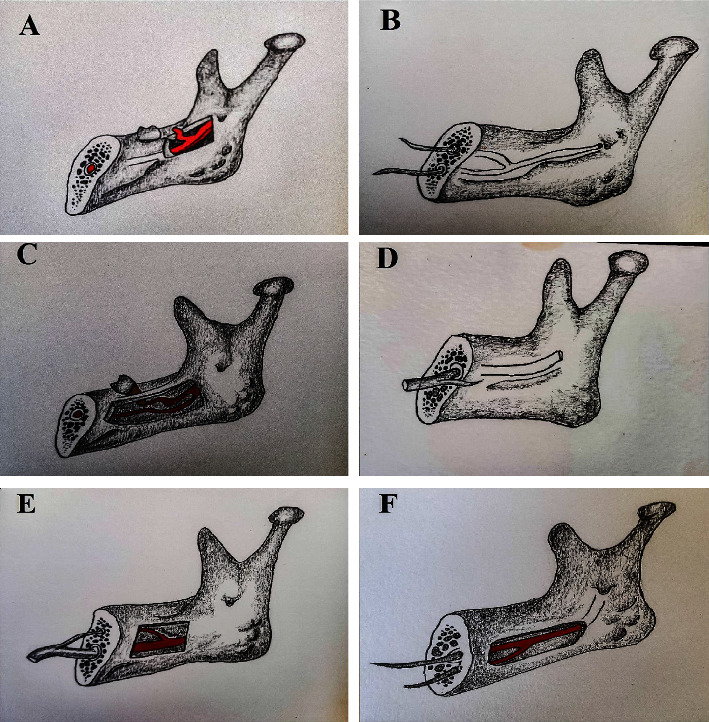
Naitoh's classification: (a) retromolar canal, (b) forward canal, (c) dental canal, (d) buccolingual canal, and (e) confluent canal. Extension of Naitoh's classification: (f) inferior bifid or bicanal.

In accordance with Naitoh's classification (Figure 7), from largest to smallest, retromolar canal accounted for 6.2% (95% CI:4.5%–8.2%) (range: 1%–17%) (*Q* = 813.27, *p*=0.001, *I*^2^ = 97%), forward canal accounted for 4.7% (95% CI: 2.9%–6.9%) (range: 0%–18%) (*Q* = 1134.66, *p*=0.0001, *I*^2^ = 98%), dental canal accounted for 2.8% (95% CI: 1.7%–4.1%) (range: 0%–21%) (*Q* = 633.58, *p*=0.0001, *I*^2^ = 97%), inferior bifid canal accounted for 2.2% (95% CI: 1.2%–3.5%) (range: 1%–4%) (*Q* = 15.51, *p*=0.008, *I*^2^ = 68%), and buccolingual canal accounted for 0.8% (95% CI: 0.4%–1.4%) (range: 0%–8%) (*Q* = 388.96, *p*=0.0001, *I*^2^ = 95%), respectively.

Of these, 8.5% (95% CI: 5.4%–12.2%) (range: 0%–100%) (*Q* = 141.33, *p*=0.0001, *I*^2^ = 87%) of BMCs were confluent or rejoined with the main mandibular canal.

#### 3.5.7. Norje's Classification (Hemi-Mandible-Level Prevalence)

According to Norje's classification, Norje ΙΙ canal revealed 7.1% (95% CI: 3%–12.8%) (range: 4%–13%) (*Q* = 20.371, *p*=0.0001, *I*^2^ = 90%), Norje ΙV canal revealed 1.8% (95% CI: 0%–5.9%) (range: 0%–5%) (*Q* = 37.874, *p*=0.0001, *I*^2^ = 95%), and Norje Ι canal revealed 1% (95% CI: 0%–3.6%) (range: 0%–4%) (*Q* = 23.789, *p*=0.0001, *I*^2^ = 92%) from highest to lowest.

#### 3.5.8. Langlais's Classification (Patient-Level Prevalence)

According to Langlais's classification, Langlais Ι canal demonstrated 1.7% (95% CI: 10%–25%) (range: 0%–2%) (*Q* = 3.291, *p*=0.349, *I*^2^ = 9%), Langlais ΙΙ canal demonstrated 1.1% (95% CI: 0%–3.5%) (range: 0%–3%) (*Q* = 30.884, *p*=0.0001, *I*^2^ = 90%), and Langlais ΙΙΙ canal demonstrated 0% (*Q* = 0.42, *p*=0.94, *I*^2^ = 0%) by the descending order.

#### 3.5.9. BMC with Two Mandibular Foramina (Hemi-Mandible-Level Prevalence) (Norje ΙΙΙ or Langlais ΙV)

**Figure 8:**
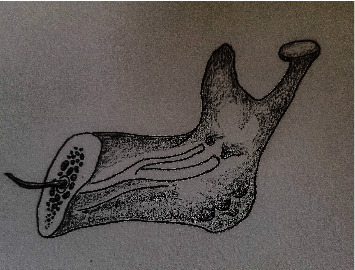
Bifid mandibular canal originated from two mandibular foramina (Norje ΙΙΙ or Langlais ΙV).

0.1% (95% CI: 0%–0.2%) (range: 0%–0.4%) (*Q* = 5.827, *p*=0.443, *I*^2^ = 0%) of BMCs originated from two mandibular foramina (Figure 8).

### 3.6. Sensitivity Analysis

#### 3.6.1. Studies Which Underwent Reliability Tests before Examining CBCT Image

Sixteen studies [34–36, 41, 42, 44, 46, 49, 50, 52, 58, 64, 65, 67, 68, 70] exhibited reliability test before examining CBCT image. With regard to patient-level prevalence of BMC, the total number of patients in these studies was 9093 of which 1384 had BMCs. The pooled patient-level prevalence of BMC was 21.8% (95% CI: 14.4%–30.3%) (range: 3%–67%) (*Q* = 1216.72, *p* < 0.05, *I*^2^ = 99%) by the random effect model. By comparing the result of objective Ι, there was a significant patient-level prevalence of BMCs in the studies having undergone the reliability test before CBCT examinations (chi-square statistic: 13.5814, *p* value = 0.000228).

Nineteen studies [13, 22, 34–36, 41, 42, 44, 49, 50, 52, 58, 63–65, 67, 69, 70] underwent reliability test and reported hemi-mandible-level prevalence of BMCs. Total count of hemi-mandibles in the studies was 17862 of which 1831 had BMCs. The combined hemi-mandible-level prevalence of BMC was 15.3% (95% CI: 10%–21.5%) (range: 2%–46%) (*Q* = 1841.474, *p* < 0.05, *I*^2^ = 99%) in the meta-analysis of the random effect model. By comparing the findings of objective ΙΙ, there was a significant hemi-mandible-level prevalence of BMCs in the studies which underwent the agreement test formerly (chi-square statistic: 33.005, *p* value = 0.00001).

### 3.7. Moderator Analysis

#### 3.7.1. Moderator Effect of Voxel Size on Prevalence of BMC

Out of forty included studies, 20 studies [13, 18, 33–35, 37, 44, 46, 47, 49, 50, 52, 53, 56–58, 63, 65, 66, 70] reported the value of voxel size. Unfortunately, the adequate amount of data for secondary data analysis was supplied by 16 studies [13, 18, 33–35, 44, 46, 47, 49, 50, 56–58, 63, 66, 70].

**Figure 9:**
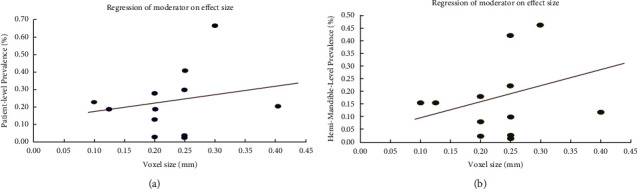
Moderator effect of voxel size of CBCT on (a) patient-level prevalence and (b) hemi-mandible-level prevalence of BMC.

Thirteen studies [34, 36, 37, 39, 41, 44, 45, 47, 48, 51, 52, 63, 66] reported patient-level prevalence of BMC for this moderator analysis. The number of patients was 6470. The reported voxel size of CBCT ranged from 0.1 to 0.4 mm. By the random effect model, there was no significant effect of voxel size on the patient-level prevalence of BMC (Figure 9(a), Table 3).

Fifteen studies [13, 18, 33–35, 44, 47, 49, 50, 56–58, 63, 66, 70] had reported hemi-mandible-level prevalence of BMCs for the moderator analysis. The total number of hem-mandibles was 12522. By the random effect model, there was a significant positive association between voxel size and hemi-mandible-level prevalence of BMC (Figure 9(b), Table 4).

### 3.8. Publication Bias

**Figure 10:**
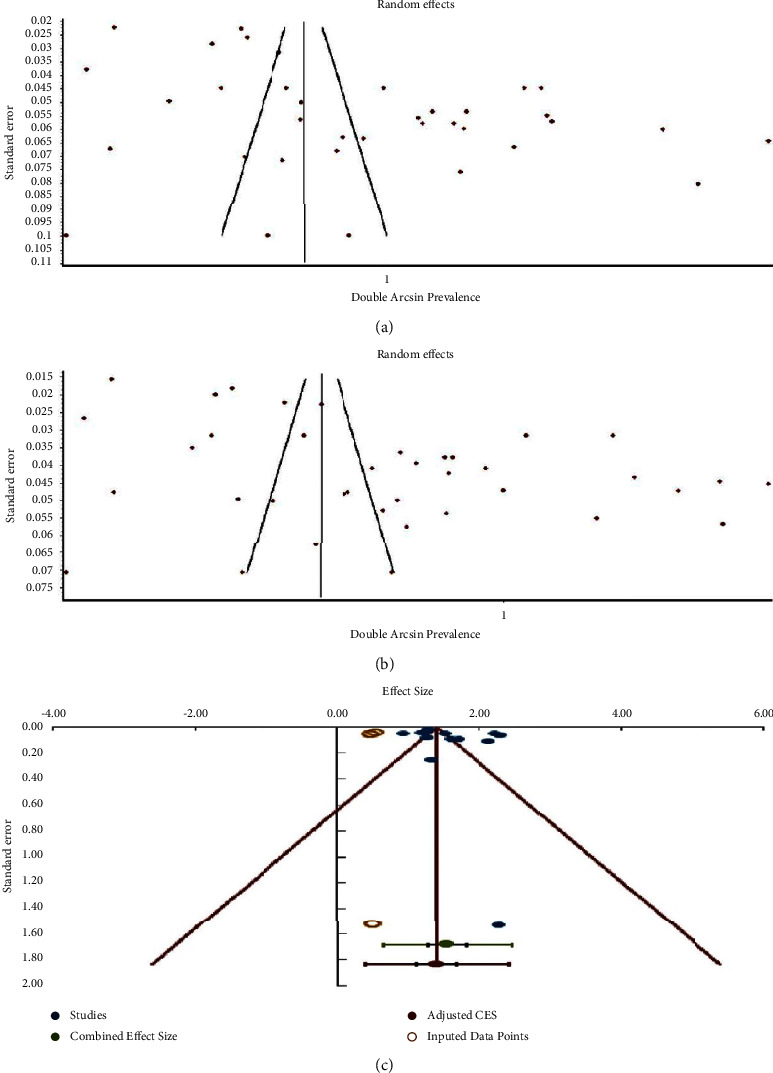
Funnel plots of (a) patient-level prevalence of BMC, (b) hemi-mandible-level prevalence of BMC, and (c) diameter of BMC adjusted by the trim-and-fill method.

Test for publication bias indicated that funnel plot asymmetry was found in objective Ι (patient-level prevalence of BMC) and objective ΙΙ (hemi-mandible-level prevalence of BMC) (Figures 10(a) and 10(b)). These figures illustrated the absence of studies at right-hand top of the plots. “Major asymmetry” for both investigations was also detected in Doi plots. LFK indexes were 2.66 for Ι and 2.78 for ΙΙ.

“No asymmetry” of the plot was seen in objective ΙΙΙ bilateral symmetrical distribution of BMC. This figure showed symmetrical distribution of the included studies. −0.92 was detected as LFK index for such case.

Publication bias was not found in the meta-analysis of the pooled estimated length of BMC (objective ΙV). On the other hand, it was investigated in the analysis of the pooled diameter of BMC (objective ΙV). The trim-and-fill test confirmed that three studies were needed to be filled on the left side of the funnel plot (Figure 10(c)).

## 4. Discussion

### 4.1. Summary of Main Findings

Resulting from the calculation, more than 20 out of hundred patients undergoing computed tomography examinations had BMCs. Those BMCs penetrated into 14% of hemi-mandibles. Of the patients having BMCs, over 23% exhibited bilateral distribution of such anatomy in the mandible.

Patient-level prevalence of BMCs ranged from 1% among people in Lucknow of India [18], 2% in Brazilians [66], and 3% in both Rasht population of Iran [56] and Samsun people of Northern Turkey [58] to 54% of Alexandria Egyptians [36] and 58% in Taiwanese Chinese in New Taipei City [55], and the peak was 67% in North Cyprus of Turkey [44].

At the hemi-mandible level, the prevalence of BMCs ranged from 1% in both Indian [18] and Brazilian populations [66] and 2% in both Turkish [58] and Iranian populations [56] to 42% in both Taiwanese [55] and Eastern Anatolia population of Turkey [34] and 43% in Cairo population of Egypt [63], and the climax was 46% in Northern Cyprus population of Turkey [44].

We emphasize that the extreme variations were seen in the Turkish populations at both patient level and hemi-mandible level.

Symmetrical occurrence of BMCs ranged from 0% in Brazilian population [38] and 2% in Pathum Thani people of Thailand [35] to 50% in Alexandria Egyptians [36] with the highest occurrence of 69% in Shenzhen population of China [59]. We notice that although Turkish populations were involved in scoring the upper tier of both patient-level and hemi-mandible-level occurrence of BMC, their constitution was almost 40% in the case of bilateral symmetry [44], ranking after China.

The accessory canals of BMCs lengthened to more than twelve millimeters in the populations of the included studies in our meta-analysis. The mean lengths of these accessory canals ranged from 7.1 mm in the Spanish patients at University of Santiago de Compostela [70] to 16.9 mm in South Korean population.

Specifically, forward, retromolar, and buccolingual canals took the longest length in Yemeni [41], South Korean [40], and Spanish [70] populations, out of other classes of Naitoh's classification. Some investigators figured out that accessory canals of BMCs were longer in premolar region than in molar [63].

On average, the canals widened to over 1.5 millimeters in diameter in the populations of the selected primary studies. The mean diameters of these accessory canals ranged from 0.9 mm in Taipei population [52] to 2.28 mm in Yemeni people of Sanaa City [41].

Comparatively, retromolar and dental canals occupied the widest, among the categories of Naitoh's classification, in South Korean [40] and Yemeni [41] populations. The accessory canal with large diameter was more prone to be detected in OPG [70]. One study mentioned that the caliber of vessel being more than one millimeter allows blood flow to flow at three milliliters per second [71]. The diameter may broaden to 3.4 millimeters in some cases [47].

### 4.2. Subgroup Analysis

The occurrence of BMCs was more exaggerated in male patients than females significantly. 6 restricted studies from Taiwan [52], Turkey [49], Tamil Nadu of India [45], Brazil [38], and Spain [70] expressed more significant prevalence of BMCs in male patients than females. Female predominance over male in the prevalence was shown only in 2 studies conducted in Turkey [39] and India [37]. The remaining studies had non-significant effect between the two sexes [33, 35, 40–44, 47, 50, 51, 54, 56, 57, 60, 61, 64, 66, 68]. We postulate that due to a huge amount of sample size in the meta-analysis, a tiny effect size (proportion) may become significant.

BMCs were more commonly found in the right half of the mandible than the left. But, a limited number of studies had right side predominance of BMC specifically in Taiwanese [55] and Turkish Istanbul populations [39]. Left side dominance of such morphology was detected only in Milan people of Italy [67]. This subgroup effect has never been identified significantly in the rest of the studies [36, 37, 42–45, 49–52, 56–58, 60, 61, 64–66, 69, 70]. We suggest the biased distribution may result from the increase in sample size of the meta-analysis, previously mentioned in sex difference.

A quarter of Europeans, nearly one-fifth of Asians, and one in ten of American population had BMCs. Subsequently, this anatomy tunneled in approximately 33% (Africa), 17% (Europe), more than 13% (Asia), and over 7% (America) of hemi-mandibles across the world. However, there were only two studies [36, 63] conducted in Egypt. This should not be considered as a true representative of all African nations.

Also, patient-level data from Africa and Oceania can never be accessed in this review.

At the patient level, the prevalence of BMCs by Naitoh's classification was 6.2% greater than Norje's classification and 11% more common than Langlais's categories. At the hemi-mandible level, the prevalence of such bifid anatomy classified by Naitoh's classification was 5.6% more frequent than Norje's classification and 15.3% more abundant than Langlais's classification.

As a result, BMC was enumerated as more abundant proportion in categories of Naitoh's classification than the other two classifications. We think that CBCT technology was progressively advanced in recent decade immediately after Naitoh and colleagues had used CBCT and invented their classification system. Collectively, this classification counted an additional coronal section of computed tomography image in contrast to other classifications. Buccolingual type of Naitoh's classification can be detected in this section.

With regard to Naitoh's classification, we estimated that retromolar canal infiltrated into more than 6%, forward canal infiltrated into nearly 5%, dental canal approximately infiltrated into 3%, and buccolingual canal infiltrated into less than 1% of the hemi-mandibles.

Most of the studies [33, 34, 40, 42, 50, 51, 58–60, 67, 69] that used Naitoh's classification stated retromolar canal as the most prevalent one. Secondly, 10 studies [36, 39, 41, 43, 44, 53, 54, 57, 65, 70] verified forward canal as the most common. 2 studies [37, 45] of Indian populations and 1 of Egypt [63] defined dental canal as the most numerous. Buccolingual canal was not detected in 5 studies [41, 42, 50, 65, 69]. Interestingly, dental canal was not found in one Chinese study [41, 42, 50, 65, 69]. Dominance and recession of each and every class of Naitoh may be overwhelmed by different ethnicities.

Additionally, inferior bifid canals also known as bicanals accounted for 2.2% in our meta-analysis. Both Elnadoury [36] and Shen [48] reported this anatomy up to more than 4%. Although Saket [63] had not reported complete picture of BMCs, he presented the picture of inferior bifid with confluence type in his article. Of the accessory mandibular canals classified by Naitoh, 8.5% rejoined into main mandibular canal (confluent type) in our meta-analysis.

According to Norje's classification, Norje ΙΙ, ΙV, and Ι displayed 7%, nearly 2%, and 1%, respectively.

By means of Langlais's classification, Langlais Ι, ΙΙ, and ΙΙΙ demonstrated nearly 2%, more than 1%, and occupied null value. Retromolar canal in Naitoh's classes are similar to Norje ΙV and Langlais Ι, forward being coincident with Norje Ι, forward confluent resembling Langlais ΙΙ, dental canal comparable with Norje ΙΙ, and the contribution of forward confluent and retromolar resembling Langlais ΙΙΙ (Table 1).

Less than one percent of hemi-mandibles orchestrated two mandibular canals originating from two separate mandibular foramina (Langlais ΙV or Norje ΙΙΙ). Although most of the investigators [33–37] had not presented such type of anatomy, some [48] reported that 6% of accessory canal of BMCs drained outside the mandible by separate foramina openings.

### 4.3. Sensitivity Analysis

In the sensitivity analysis, after excluding the studies which did not perform inter or intra-examiner reliability tests, both patient-level and hemi-mandible-level prevalence of BMC escalated to more than 1%, respectively. 32% to 99% agreement within or between examiners, in detecting the prevalence of BMCs, was reported [13, 18–34, 36, 41, 42, 44, 46, 49, 50, 52, 58, 63–65, 67–70].

We hypothesize that inter or intra-rater reliability test before CBCT examinations could have a slight positive influence on prevalence of BMC. Because of half of the selected studies not having undergone the test, the pooled estimate of this meta-analysis may be underestimated.

### 4.4. Moderator Analysis

By undergoing moderator analysis between voxel size of CBCT and hemi-mandible-level prevalence of BMCs, Z value exceeded 1.96 and 0 was not included in the confidence limits of slope (Table 4). This indicates the significant positive association between hemi-mandible-level prevalence of BMC and voxel size of CBCT. The voxel sizes were reported ranging from 100 to 400 *µ*m [13, 18, 33–35, 37–44, 46, 47, 49, 50, 52, 53, 56, 58, 63, 65, 66, 70]. Although smaller voxel size potentiates the resolution of CBCT, the reverse can be proved by the analysis. However, the reviewers could not explain why the studies having reported high degree of prevalence of BMCs used larger voxel sizes.

### 4.5. Agreement and Disagreement with the Previous Reviews

Haas and colleagues [23] previously investigated that patient-level prevalence of BMC was 16.25% by CT or CBCT. This is obviously lower than the findings of our analysis. The pooled prevalence of BMCs in both in vitro studies and the studies, which used OPG as investigation method, comprised 6.5% and 4.2% in Haas's meta-analysis [23]. Also, these disagreements may be due to dissimilarity between research methods used in selected studies.

Valenzuela-Fuenzalida and coworkers [22] found 57% of BMCs in more than 4000 mandibles. This is superior to the finding of our analysis displaying more than 20 percent of more than 17000 mandibles. The previous analysis [22] manipulated not only CBCT studies but also cadaver studies and the studies that used dry human skulls. So, we make the assumption that the proportions of BMCs may be inflated due to smaller sample size of primary studies, distortion of specimen, and fragility of dry mandibular bone.

Ngeow and Chai [12] showed patient-level prevalence of BMCs from 0.05% to 69%. The range was complementary with our finding which ranged between 1% and 67%. They also observed mean length of accessory canal of BMCs ranging from 7.1 mm to 16.9 mm. This is in agreement with our analysis again. The previous review also pointed out that the mean diameters of the accessory canal ranged from 0.9 mm to 2.2 mm. We identified that our range (0.9 mm–2.28 mm) was in turn in agreement with the previous findings.

In the review of Shah and Mehta [24], retromolar canal comprised 3.2% to 93.5% of dry mandibles. This range was five times wider than ours. As a result, we think that restriction to this subtype of BMC, dry bone sample, and methodological diversity may greatly overwhelm the prevalence of retromolar canal.

By comparing other aberrant anatomy of the mandible, Muinelo-Lorenzo et al. [11] summarized that accessory mental foramina were detected nearly 8% at the patient level. Additionally, Mishra and associates [11] showed that anterior loops of mental nerve were seen up to 41% at the subject level. So, we recognize that BMCs were found more than twice of the accessory mental foramina and less than half of the anterior loops at the individual level. However, we did not attempt to analyze the associations between these aberrant anomalies and BMCs in this review.

Additionally, Mishra's group [11] also stated that the average length of anterior loops ranged from 1.1 mm to more than 8 mm. The upper limit of anterior loop's length could be overlapped with the lower bound of mean length of accessory canal of BMCs.

Castro and fellows [25] reviewed the classifications of BMCs. They especially concentrated on the radiographic methods used in classification systems, two or three-dimensionally. They also outlined locations of BMCs at mandibular ramus or at body of the mandible. Contrastively, from our standpoint, we emphasize on units of analysis at patient level or hemi-mandible level and similarity among different classifications (Table 1).

### 4.6. Risks of Bias

Although overall average JBI score of all included studies in this meta-analysis had been 54.69% (moderate risk of bias), some limitations were seen at the domains of sampling characteristics, sample size calculation, population coverage, reliability test, and outcome reporting.

The worst domain that seems to be suffering from risk of bias was sample size calculation. The prior estimation of sample was never attempted in 90% of the included studies (Figure 3). Also, 65.85% of the studies used the records within inadequate time frame and took the sample from single centers or university, not from several centers. This may lead to under-coverage of target population and could not be true representative of such population.

Consequently, 60% of the eligible studies neglected population characteristics (age, gender, and ethnicity) to record thoroughly. 50% of the studies did not obtain inter or intra-examiner agreement test before CBCT examinations. Additionally, 45% of the studies did not report patient-level and hemi-mandible-level prevalence of BMCs, their bilateral distribution, and other suitable outcomes sufficiently. These factors could be prone to misclassification of BMCs and incomplete outcome reporting.

### 4.7. Heterogeneity

Heterogeneity grew considerably in case of the meta-analyses at patient-level prevalence, hemi-mandible-level prevalence, and bilateral symmetrical distribution of BMCs.

To explore the source of heterogeneity, we conducted multiple subgroup analyses. By doing so, we identified some heterogeneities that originated from the classifications used in primary studies. When we had categorized the studies into their corresponding classifications, the meta-analyses demonstrated some relief from heterogeneity.

For Norje's classification, *I*^2^ statistic decreased from 98% to 51% at the patient-level prevalence of BMCs and from 99% to 83% at hemi-mandible-level prevalence.

For Langlais's classification, *I*^2^ dropped from 98% to 73% at patient level and from 99% to 87% at the hemi-mandible level of BMCs, respectively. Across the different classes of Langlais classification, the parameter decreased from 98% to 9% in Langlais Ι and to 0% in Langlais ΙΙΙ at the patient-level prevalence of BMCs.

At the hemi-mandible-level prevalence of BMCs, *I*^2^ statistic of heterogeneity fell from 99% to 0% in case of inferior bifid type of BMCs and to 87% for the confluent type BMCs.

No substantial loss of heterogeneity was found in Naitoh's classification.

For African continent, *I*^2^ reduced from 99% to 8% at hemi-mandible-level prevalence of BMCs. In such case, we speculate that it may be due to the scarcity of evidences in the African nations.

Finally, we conclude that the heterogeneity in prevalence of BMCs can be partly explained by the different classification systems used in selected primary studies.

### 4.8. Imperfections of BMC's Classifications

Among BMC's classifications, Norje's [36] and Langlais's [37] categorizations were based upon two-dimensional X-ray examinations, while Naitoh and coworkers [35] had investigated by viewing three-dimensional computed tomography. The most distinguished feature between the latter and former is the inclusion of coronal view in Naitoh's classification.

This additional view integrates the buccolingual type of BMCs in Naitoh's classes, which is never found in both Norje's and Langlais's contributions (Table 1). On the other hand, two mandibular foramina types were not seen in Naitoh's classification, although they had been previously set in Norje's and Langlais's classifications.

Lateral lingual and median lingual canal stated in other studies [72] could be misinterpreted as buccolingual type of BMC. Dental canal of Naitoh's classification can lose its identity after extraction of corresponding tooth. So, it may be misunderstood as Naitoh's retromolar and forward types. We cannot mention precisely how nutrient canals and edentulous condition influence the classification of BMCs in CBCT image.

Additionally, plexus form [73], curved or horizontal or vertical typed retromolar canal [74], hypertrophic [71], double-confluent type [25], superior canal [70], ramus canal [75], canal of mandibular coronoid process [76], condylar canal [77], the accessory canal associated with dental inflammation [78], BMC with separate mandibular foramina [48], inferior alveolar nerve bifurcated or perforated by maxillary artery before entering mandibular foramen [7], lateral lingual canal [72], median lingual canal [72], V-type retromolar canal [50], fork-like trifid [33], and canal draining at temporal crest [79] are all implicated and confused with terminology and conditions of BMCs. Retromolar canal was not counted as BMC and set as a separate class by some investigators [22]. Specifically, plexus type of BMC was found together with inflammation [78]. It seems to be the association of nerve growth, bone resorption, and inflammation. Moreover, presence of BMCs was positively associated with bony area of the mandible [52].

Additionally, BMCs were also associated with accessory mental foramina in 73.68% of Brazilians [46]. However, this class was not included in most of the classifications [35–37].

The questionable content and construct validity lead to imperfections of the classifications.

### 4.9. Content of Accessory Canal of BMC

Vein, artery, nerve, and lymphatic drainage are major constituents of the main mandibular canal. However, in place of the assembly of vein, artery, nerve, and lymphatic drainage, only one large venous vessel supplying base of mandible [3] or nutrient vessels [80] or bone marrow [20] or multiple osteoporosis cavities [81] or proximal branching of mental nerve at the entrance of mandibular foramen [82] or remaining nerve plexus [83] of edentulous mandible can be present in the accessory mandibular canals of BMCs of cadaver sample. These structures can correspond to be radiolucency in computed tomography images.

Unfortunately, strictly bony radiographic architecture of the accessory canal can be seen in CBCT bone-contrast image. Soft tissue content of this additional canal cannot be found in the image.

### 4.10. OPG versus CBCT versus MRI in Detecting BMCs

Only 16.67% of BMCs investigated in MRI were found in CBCT [19]. Sequentially, 11% to 76% of BMCs [13, 17, 18] detected in CBCT image were also seen in OPG. Occasionally, radio-opaque mylohyoid line on lingual plate of mandible was misinterpreted as BMC (false positive) in OPG image [17].

As a result, MRI is the current gold standard method in observing not only BMC but also its contents. Blood vessels and nerve can be well differentiated by viewing signal intensities of MRI because vein exhibits more intensified features than nerve in MRI image. So, even VANL assembly can be detected in MRI [19].

Unfortunately, because of MRI being soft-tissue contrast, two mandibular foramina of BMCs may not be seen accurately in this image. So, some investigators [19] advised that they should be confirmed by CBCT, which is hard-tissue contrast, in this case.

Bifid mandibular nerves may not always occupy two mandibular canals. The mandibular canal wall is mostly formed by facing trabecular bony plates inside while their bony pillar orienting outside [84]. This pillar-plate orientation could be destroyed by bone diseases. Furthermore, this proves that the mandibular canal wall does not possess specific compact or specialized bony structure in nature although, not rarely, radiopaque line is seen along the course of this canal in formal radiographic examinations.

To the best of our knowledge, we conclude that bifid mandibular nerves may be present even in a single hollow of bone cavity. Also, this could be missed during routine radiological examination.

### 4.11. Publication Bias

Three studies [54, 59, 60] from mainland China were translated from Chinese to English, 1 study [42] and 1 thesis [65] from Peru were translated from Spanish to English, and 1 study [69] from Spain was translated from Spanish to English.

Although 5 studies of languages other than English [42, 54, 59, 60, 69] and 1 thesis (gray literature) [65] were included in this meta-analyses, major publication bias was subjectively seen in both patient-level and hemi-mandible-level prevalence of BMCs and pooled diameter of accessory canals of BMCs.

### 4.12. Future Studies

In spite of progressive number of evidences investigating BMCs being found, pooled sensitivity and specificity of CBCT in comparison with gold standard MRI in detecting this anatomy will be needed to be questioned and pooled.

## 5. Conclusion

Generally, 20.7% of patients seeking computed tomography examinations and 14.3% of hemi-mandibles displayed BMCs. Nearly 23% of those patients exhibited bilateral distribution of such specific anatomy. On average, the accessory canal of BMCs lengthens up to 12.14 millimeters and widens to 1.54 millimeters. Sexual dimorphism towards male gender and right-sided predominance of the canal were seen together with high statistical power and sample size of the meta-analyses. Europeans were found to be the population in which BMCs were mostly investigated all over the world. Usage of Naitoh's classification and reliability tests may escalate the proportion of BMCs. We uncovered one unexplainable reason in which voxel size of CBCT may have positive correlation with prevalence of BMCs with no regard to considering other resolution parameters.

## Figures and Tables

**Table 1:** Commonality among Naitoh's, Norje's, and Langlais's classifications.

Naitoh's classification	Langlais's classification		Norje's classification
Forward	—		Ι
Forward confluent	ΙΙ	ΙΙΙ	—
Retromolar	Ι		ΙV
Dental	—		ΙΙ
Buccolingual	—		—
—	VΙ	Two mandibular foramina	ΙΙΙ

**Table 2:** Author names, population, country, number of patients, number of mandibular canals, geographic locations, conditions defined by the primary studies, CBCT setting, and study design.

No	Study name	Population	Country	Number of patients	Number of hemi-mandibles	Age of the patients (years)	Geographic locations	Terminology of the condition	CBCT setting	Study design
1	Neves et al.^*∗*^ [18]	Brazilian population	Brazil	127, M: 55, F: 72	254	18–61	America	Bifid mandibular canal	i-CAT, 120 kVp, 8 mA, voxel size 0.25 mm, FOV 8 cm	Cross-sectional, analytical, and retrospective
2	Rashsuren et al. [33]	South Korean population	South Korea	500, M: 290, F: 210	755	10–>50	Asia	Bifid mandibular canal	Alphard VEGA CBCT, 80 kV, 8 mA, voxel size 0.1 mm	Cross-sectional, descriptive, and retrospective
3	Dedeoglu and Duman [34]	Turkish Eastern Anatolia population	Turkey	332	501	19–71	Europe	Bifid mandibular canal	NewTom 5 G (Verona, Italy), 110 kV, maximum 20 mA, FOV 18 × 16, 15 × 12 and 12 × 8, voxel sizes 0.2, 0.25, and 0.3 mm	Cross-sectional, descriptive, and retrospective
4	Karnasuta et al. [35]	Thai population	Thailand	248, M: 98, F: 150	441	20–82	Asia	Bifid mandibular canal	I-CAT classic CBCT, scanner, 120-kV, 24-mA, 13-cm FOV, voxel size 0.4	Cross-sectional, descriptive, and retrospective
5	Elnadoury et al. [36]	Egyptian population	Egypt	278	530	≥18	Africa	Bifid mandibular canal	I-CAT next generation 120 kVp, 5 mA, 0.25 mm voxel size	Cross-sectional, descriptive, and retrospective
6	Chanda et al. [37]	Indian population	India	100, M: 52, F: 48	200	15–72	Asia	Accessory mandibular canal	ProMax 3 D, 90 kVp, 10 mA, and voxel size ranged, from 0.2 to 0.4 mm, FOV- variable	Cross-sectional, descriptive, and retrospective
7	Villaça-Carvalho et al. [38]	Brazilian population	Brazil	300, M: 178, F: 122	600	25–87	America	Bifid mandibular canal	Classic I-Cat, 120 kVp, 5 : 07 mA	Cross-sectional, descriptive, and retrospective
8	Okumuş and Dumlu [39]	Turkish population	Turkey	500, M: 250, F: 250	1000	14–79	Europe	Bifid mandibular canal	Planmeca ProMax 3D, 90 kV, 12 mA	Cross-sectional, descriptive, and retrospective
9	Kang et al. [40]	Korean population	South Korea	1933, M: 884, F: 1049	1933	13–93	Asia	Bifid mandibular canal	-PSR-9000 N, 80 kVp, 10 mA, FOV 41 × 41 mm—Alphard Vega 3030, 80 kVp, 5 mA, FOV 51 × 51 mm	Cross-sectional, descriptive, and retrospective
10	Qaid et al. [41]	Yemeni population	Yemen	400, M: 238, F: 162	400	18–70	Asia	Bifid mandibular canal	PaX-Flex3D, P2, FOV = 13 cm	Cross-sectional, descriptive, and retrospective
11	Quispe-Huarcaya et al. (**T**) [42]	Peruvian population	Peru	1497, M: 527, F: 970	2994	10–89	America	Bifid mandibular canal	CBCT, 10–40 mA, 85 kVp,	Cross-sectional, descriptive, and retrospective
12	Yang et al. [43]	Shanghai Chinese population	China	280, M: 142, F: 138	560	18–78	Asia	Bifid mandibular canal	Planmeca ProMax 3D CBCT, FOV 16 × 16 cm, 90 kVp, 2 mA	Cross-sectional, descriptive, and retrospective
13	Orhan et al. [44]	Turkish adult population	Turkey	242, M: 109, F:133	484	17–83	Europe	Bifid mandibular canal	NewTom 3G, 120 kVp, 3–5 mA, FOV 9-inch, 0.3 mm voxels size	Cross-sectional, descriptive, and retrospective
14	Nithya and Aswath [45]	Indian population from Tamil Nadu	India	203, M: 125, F: 78	406	>15	Asia	Bifid mandibular canal	MyRay SkyView, CBCT	Cross-sectional, descriptive, and retrospective
15	Oliveira-Santos et al. [46]	Brazilian population	Brazil	100, M: 41, F: 59	200	≤20–>60	America	Bifid mandibular canal	Scanora 3D^®^, voxel size 0.2 mm,	Cross-sectional, descriptive, and retrospective
16	Kuribayashi et al. [47]	Japanese population	Japan	252, M: 94, F: 158	301	18–74	Asia	Bifid mandibular canal	3DX multi-image, micro CT, 80 kVp, 2 mA, 0.125 mm voxel size	Cross-sectional, descriptive, and retrospective
17	Shen et al. [48]	Taiwanese population	Taiwan	308	616	12–85	Asia	Mandibular bifid canal	KaVo 3D eXam, multi-slice, CT 120 kVp, 5–300 mA,	Cross-sectional, descriptive, and retrospective
18	Laçin et al. [49]	Turkish population	Turkey	350, M: 178, F: 172	700	18–65	Europe	Bifid mandibular canal	NewTom 5G CBCT, 1–20 mA, 15×12 FOV, 0.2 mm voxel size	Cross-sectional, descriptive, and retrospective
19	Zhang et al. [50]	Northern Chinese population	China	1000 M: 341, F: 659	2000	18–60	Asia	Bifid mandibular canal	NewTom VGi, 110 kV, automatic mA, voxel size of 200 *μ*m, FOV of 15 × 12 cm	Cross-sectional, descriptive, and retrospective
20	Zhou et al. [51]	Chinese population	China	321, M: 150, F: 171	642	8–80	Asia	Bifid mandibular canal	KaVo 3D eXam CBCT	Cross-sectional, descriptive, and retrospective
21	Fu et al. (**Ć**) [52]	Taiwanese population	Taiwan	173, M: 76, F: 97	346	14–85	Asia	Bifid mandibular canal	64-slice multi-detector CT system, 120 kV, 300 mA	Cross-sectional, descriptive, and retrospective
22	Komal et al. [53]	Lucknow Indian population	India	100, M: 53, F: 47	200	Mean age, M: 40.96 ± 14.77, F: 41.85 ± 14.36	Asia	Bifid mandibular canal	i-CAT CBCT, 0.125/0.2 mm voxel, 270 kV and 10–15 mA, FOV 14 × 6 cm and 8.5 × 6 cm and 0.125 × 0.125 × 0.125 mm^3^	Cross-sectional, descriptive, and prospective
23	Yi et al. (**T**) [54]	Weifang Chinese population	China	216, M: 112, F: 104	432	—	Asia	Bifid mandibular canal	Sirona Galileos CBCT, 7 mA, 85 kVp	Cross-sectional, descriptive, and retrospective
24	Shen et al. (**+**) [55]	Taiwanese population	Taiwan	154, M: 160, F: 167	308	23–85	Asia	Bifid mandibular canal	CBCT (KaVo 3D) eXam scanner, 120 kV and 5 ma, for MSCT, helical pitch of 0.531, gantry rotation time, 1 second, tube voltage, 120 kV, tube current 300 mA	Cross-sectional, descriptive, and retrospective
25	Dalili Kajan et al. [56]	Iranian population	Iran	221, M: 92, F: 129	442	—	Asia	Bifid mandibular canal	New-Tom CBCT, voxel size: 0.2–0.24 mm, FOV = 10 × 10 cm	Cross-sectional, descriptive, and retrospective
26	Freitas et al. [57]	Brazilian population	Brazil	300	600	13–87	America	Bifid mandibular canal	Classic I-Cat^®^, voxel size 0.25 mm, FOV of 13 cm, 120 kVp, 5–7 mA	Cross-sectional, descriptive, and retrospective
27	Serindere et al. [58]	Turkish population	Turkey	2000, M: 878, F: 1122	4000	18–75	Europe	Bifid mandibular canal	Galileos, 98 kVp, 25 mA, FOV 15 × 15 × 15 cm^3^, voxels 0.25 × 0.25 × 0.25 mm^3^	Cross-sectional, descriptive, and retrospective
28	Yu et al. (**T**) [59]	Chinese population	China	500, M: 305, F: 195	1000	—	Asia	Bifid mandibular canal	NewTom VGi, FOV 12 cm × 8 cm, 110 kVp	Cross-sectional, descriptive, and retrospective
29	Li et al. (**T**) [60]	Sichuan Chinese population	China	500, M: 264, F: 236	1000	18–60	Asia	Bifid mandibular canal	3D Accuitomo	Cross-sectional, descriptive, and retrospective
30	Yang et al. [61]	Hunan Chinese population	China	350, M: 120, F: 230	700	—	Asia	Bifid mandibular canal	Planmeca CBCT scanner	Cross-sectional, descriptive, and retrospective
31	Singh et al. [62]	Indian Bangalore population	India	408	816	—	Asia	Bifid mandibular canal	CBCT, 90 kVp, 6.3 mA	Cross-sectional, descriptive, and retrospective
32	El Saket et al. [63]	Egyptian population	Egypt	—	329	18–70	Africa	Bifid mandibular canal	Planmeca ProMax 3D mid CBCT, voxel size 0.4 mm, different FOVs	Cross-sectional, descriptive, and retrospective
33	Yoon et al. [64]	American population	United States	194, M: 86, F: 108	398	13–103	America	Bifurcated IAN	Sirona XG3 CBCT, 6 mA, 85 kVp, FOV 8 × 8 cm	Cross-sectional, descriptive, and retrospective
34	Cano valqi et al. (**T**, **$**) [65]	Peruvian population	Peru	1239	2478	—	America	Mandibular bifurcation	Promax 3D, voxel 200–400 *µ*m, 90 kVp, 14 mA, 10 × 10 cm, 10 × 20 cm FOV	Cross-sectional, descriptive, and retrospective
35	Casagrande et al. [66]	Brazilian population	Brazil	700, M: 252, F: 448	1400	6–82	America	Bifid mandibular canal	i-CAT, FOV 16 × 13 cm, voxel size 0.25 mm, 37.07 mA and 120 kVp	Cross-sectional, descriptive, and retrospective
36	Borgonovo et al. [67]	Italian population	Italy	500	1000	>18	Europe	Mandibular accessory canal	Kavo 3D eXam, 120 kV, 5 mA	Cross-sectional, descriptive, and retrospective
37	Adışen and Aydoglu [68]	Turkish population	Turkey	362, M: 152, F: 210	—	10–87	Europe	Bifid mandibular canal	I-CAT, 23 cm × 17 cm FOV, 18.54 mA, 120 kVp	Cross-sectional, descriptive, and retrospective
38	Vranckx et al.^*∗*^ [13]	Belgian population	Belgium	201 M: 83, F: 118	357	26.4 ± 8.6	Europe	Bifid mandibular canal	Newtom VGi evo, voxel size 0.2, kVp 110, mA 4–5	Cross-sectional, analytical, and retrospective
39	Sirera-Martin et al. (**T**) [69]	Spanish population	Spain	100	200	31–55	Europe	Bifid mandibular canal	Planmeca ProMax 3D, 90 kVp, 12 mA	Cross-sectional, descriptive, and retrospective
40	Muinelo-Lorenzo et al.^*∗*^ [70]	Spanish population	Spain	225, M: 90, F: 135	450	13–79	Europe	Bifid mandibular canal	i-CAT® model 17–19, 120 kVp, 5 ma, <0.3 mm voxel size	Cross-sectional, analytical, and retrospective

^*∗*^Comparison between CBCT and OPG (analytical study design); **T**, translation; **$**, thesis; **+,** multi-slice computed tomography + cone beam computed tomography; **Ć**, computed tomography.

**Table 3:** Moderator analysis between voxel size of CBCT and patient-level prevalence of BMC.

	*B*	SE	CI LL	CI UL	*β*	*Z* value	*p* value	
Intercept	0.112	0.07	−0.02	0.28		1.75		0.08
Slope	0.49	0.30	−0.16	1.15	0.2	1.63		0.102

*B*, the rate of change per unit time; SE, standard error; CI, confidence interval; LL, lower limit; UL, upper limit; *β*, correlation coefficient ranging from 0 to 1; *Z* value, regression coefficient divided by standard error.

**Table 4:** Moderator analysis between voxel size of CBCT and hemi-mandible-level prevalence of BMC.

	*B*	SE	CI LL	CI UL	*β*	*Z* value	*p* value
Intercept	0.03	0.04	−0.04	0.11		0.89	0.373
Slope	0.64	0.14	0.33	0.94	0.36	4.47	0.0001

*B*, the rate of change per unit time; SE, standard error; CI, confidence interval; LL, lower limit; UL, upper limit; *β*, correlation coefficient ranging from 0 to 1; *Z* value, regression coefficient divided by standard error.

## Data Availability

The data supporting the findings of this study are available from the corresponding author upon reasonable request.
